# Engineered three-dimensional bioactive scaffold for enhanced bone regeneration through modulating transplanted adipose derived mesenchymal stem cell and stimulating angiogenesis

**DOI:** 10.3389/fbioe.2024.1342590

**Published:** 2024-01-26

**Authors:** Gan Wang, Yutao Cui, Yi Leng, Shouye Sun, Baoming Yuan, He Liu, Chuangang Peng, Dankai Wu

**Affiliations:** ^1^ Orthopaedic Medical Center, The Second Hospital of Jilin University, Changchun, China; ^2^ Department of Orthopaedics, The First Hospital of Jilin University, Changchun, China

**Keywords:** 3D printing, platelet-rich plasma, adipose mesenchymal stem cells, angiogenesis, bone repair

## Abstract

Titanium alloy materials are commonly used in orthopedic clinical treatments. However, conventional titanium implants usually lead to insufficient bone regeneration and integration because of mismatched biomechanics and poor bioactivities. To tackle these challenges, a porous titanium alloy scaffold with suitable mechanical properties was prepared using three-dimensional (3D) printing, and then an adipose-derived mesenchymal stem cell (ADSC) loaded platelet-rich plasma (PRP) gel was placed into the pores of the porous scaffold to construct a bioactive scaffold with dual functions of enhancing angiogenesis and osteogenesis. This bioactive scaffold showed good biocompatibility and supported cell viability proliferation and morphology of encapsulated ADSCs. Osteogenic and angiogenic growth factors in the PRP gel promoted the migration and angiogenesis of human umbilical vein endothelial cells (HUVECs) *in vitro* and enhanced osteogenic-related gene and protein expression in ADSCs, thus promoting osteogenic differentiation. After implantation into the femoral defects of rabbits, the bioactive scaffold promoted vascular network formation and the expression of osteogenesis-related proteins, thus effectively accelerating bone regeneration. Therefore, the osteogenic and angiogenic bioactive scaffold comprising a 3D printed porous titanium alloy scaffold, PRP, and ADSCs provides a promising design for orthopedic biomaterials with clinical transformation prospects and an effective strategy for bone defect treatment.

## 1 Introduction

Large segmental bone defects caused by severe trauma, bone tumors, and congenital diseases increase the implementation of bone reconstruction procedures, which have brought about not only advances in surgical techniques but also the development of implants in orthopedics (Liu et al., 2017; Sparks et al., 2020). Clinically, autologous bone transplantation remains the gold standard for bone reconstruction. Although autologous bone transplantation has a definite therapeutic effect, complications caused by donor site surgery and limited available bone mass are also an inextricable deficiency (Crouzier et al., 2011; Bez et al., 2017). As an alternative, allogeneic bone transplantation also suffers from immunologic rejection, disease transmission, and a high resorption rate (Larsen et al., 2011; Yan et al., 2019). Therefore, the development of ideal implants for large segmental defects remains a major challenge for orthopedic clinical treatment.

In recent years, several synthetic biocompatible materials, such as bioceramics, metal materials, and polymer materials, have been designed and developed as bone substitutes in response to this challenge. Among them, three-dimensional (3D) porous titanium alloy implants (PTIs) have been commonly utilized, attributable to their good biocompatibility, excellent corrosion resistance, and mechanical properties (Li et al., 2015). PTIs mimicking natural bone both morphologically and mechanically can be produced using 3D printing additive manufacturing technology. The interconnected porous structure not only avoids stress-shielding defects but also provides space for bone ingrowth (Zhang et al., 2019; Cui et al., 2021). Although these characteristics provide PTIs with a wide application range in bridging large bone defects, clinical failure can result from poor bone ingrowth and subsequent poor stability due to the inert surface of Ti alloys (Meng et al., 2016; Kelly et al., 2021; Ma et al., 2021). Therefore, improvement in promoting the osteogenic activity of PTIs for their better clinical applications is a promising research area.

Due to their self-renewal capacity, multi-directional differentiation potential, and hypoimmunogenicity, mesenchymal stem cell (MSC) transplantation promotes bone and cartilage repair in preclinical models and clinical trials (Clark et al., 2020). Therefore, a combination of MSC transplantation and porous PTIs can improve the osteogenic activity of the porous scaffold. However, without an appropriate carrier, transplanted MSCs have poor survival and retention rates at the defect site, and the majority of MSCs will die or be washed away (Levit et al., 2013; Bai et al., 2020a). Platelet-rich plasma (PRP) is a platelet concentrate prepared from whole blood that can be obtained as a platelet gel upon mixing with thrombin, providing an excellent MSC carrier (Lucarelli et al., 2003; Fang et al., 2020). In addition to providing a matrix network for MSCs, PRP gels release a variety of osteogenic and angiogenic growth factors such as platelet-derived growth factor (PDGF), insulin-like growth factor 1 (IGF-1), basic fibroblast growth factors (bFGFs), and vascular endothelial growth factor (VEGF) after activation, which can promote osteogenic differentiation in transplanted MSCs, and improve the survival rate of transplanted MSCs by promoting angiogenesis and providing nutrients (Leng et al., 2020; Jiang et al., 2021).

Bone marrow mesenchymal stem cells (BMSCs) are the most commonly transplanted MSCs for the treatment of bone defects. However, recent studies have shown that adipose-derived mesenchymal stem cells (ADSCs) have similar differentiation potential to BMSCs. ADSCs are more abundant and are much more easily obtained through a simpler, more economical, and less invasive method (Niemeyer et al., 2010; Man et al., 2012). Therefore, ADSCs are more suitable for transplantation than BMSCs.

**SCHEME 1:**
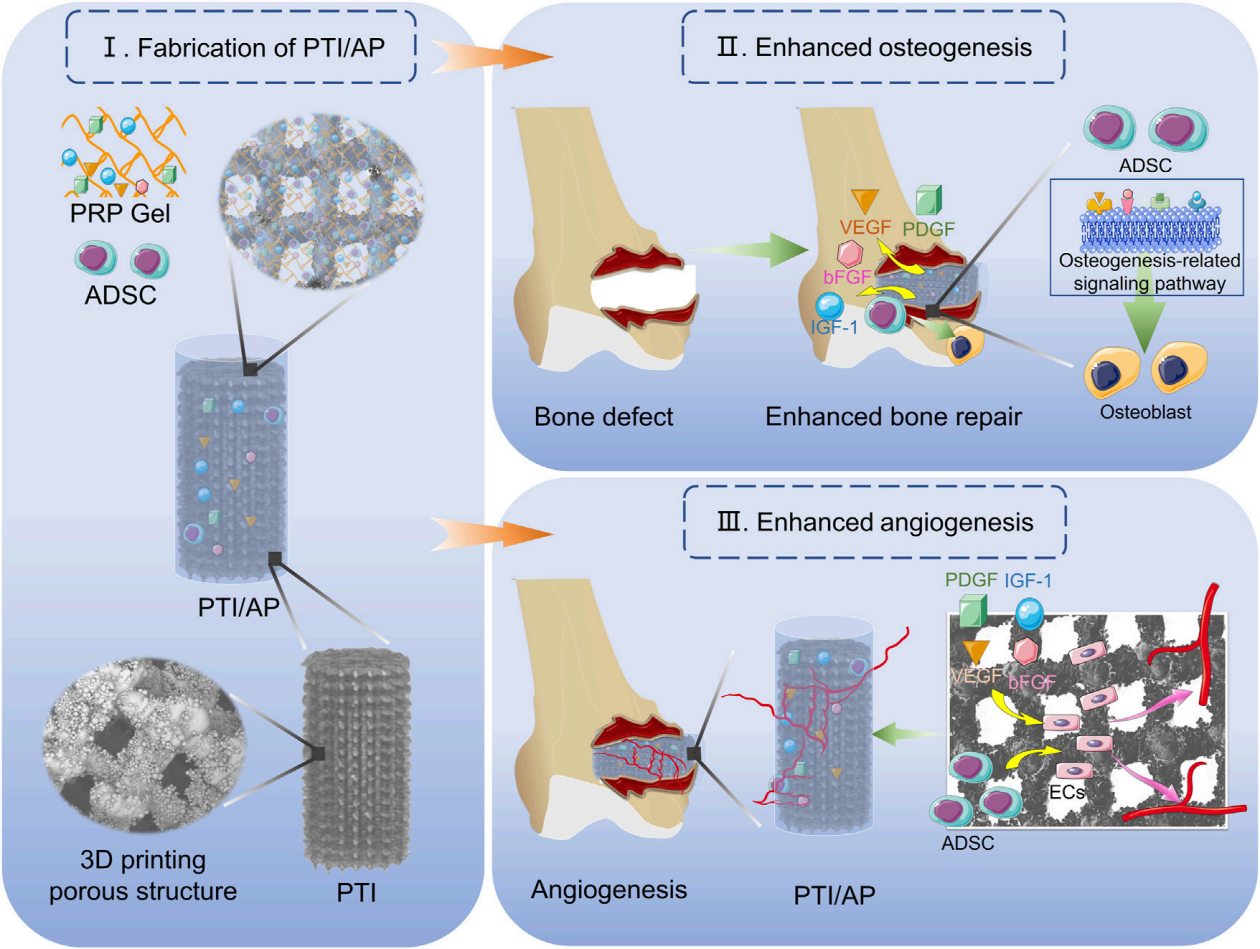
Demonstration of the construction of PTI/AP and its function of promoting bone defect repair.

In this study, we loaded ADSCs into a PRP gel matrix and placed the composite PRP gel into the PTI pores to develop a bioactive scaffold (PTI/AP). PTIs have an appropriate pore structure and mechanical properties that are similar to natural bone tissue. We studied the release of various growth factors from PTI/AP, and the impact of the bioactive scaffold on cell vitality, proliferation, osteogenic differentiation of transplanted ADSCs, and angiogenesis of vascular endothelial cells was evaluated *in vitro*. Furthermore, the bone integration efficacy of the bioactive scaffold and its promotion of bone defect repair were studied *in vivo* (Scheme 1).

## 2 Materials and methods

### 2.1 Fabrication of PTIs

A PTI with a pore size of 600 μm, strut size of 300 μm, and porosity of 70% was prepared using the electron beam melting (EBM) approach as previously described (Bai et al., 2020b). In brief, a 3D model of the PTI was designed and converted into a standard file, which was then input into the EBM system (Q10, Arcam, Sweden) for printing. Disk-shaped scaffolds with a diameter of 10 mm and a height of 3 mm were designed for *in vitro* experiments, while cylindrical scaffolds with a diameter of 5 mm and a height of 10 mm were designed for *in vivo* studies. All obtained porous scaffolds were sequentially placed in acetone, ethanol, and deionized water for ultrasonic cleaning for 15 min.

### 2.2 Preparation and characterization of PRP gel

In order to avoid the inflammatory reaction caused by leukocytes after scaffold implantation, leukocyte-poor PRP (Lp-PRP) was prepared in this study. Briefly, whole blood taken from the rabbit auricular vein was centrifuged at 200 *g* for 10 min to remove the precipitated erythrocyte. Next, the platelet-containing supernatant was subjected to a second centrifugation at 180 *g* for 10 min to remove white blood cells and residual red blood cells. Finally, a third centrifugation at 600 *g* for 10 min was performed to enrich platelets. The obtained PRP was used for subsequent experiments.

To characterize the PRP, the platelet, leukocyte, and erythrocyte contents were tested, and growth factors were detected using an enzyme-linked immunosorbent assay (ELISA) kit according to the manufacturer’s instructions.

After thrombin and 10% calcium chloride were added according to the method described by predecessors, the obtained PRP changed from solution to gel state (Cavallo et al., 2016; Wei et al., 2021). The medium containing ADSCs was mixed with the same volume of PRP. Then, thrombin and calcium chloride were added, and the PRP gel was obtained. Rheological experiments were performed to evaluate the mechanical properties of PRP gels.

### 2.3 Construction and characterizations of the PTI/AP

In order to prepare PTI/AP, a total of 100 μL of culture medium containing 2 × 10^4^ ADSCs was mixed with 100 μL of PRP according to the previous method, and the mixed solution was placed in the pores of PTI (Lu et al., 2021). Next, 10 ul of 1000 U/mL thrombin and 20 μL of 10% calcium chloride were added, and the PRP gel was obtained *in situ* inside the PTI.

The surface morphology of PTI/AP was detected using a scanning electron microscope (SEM). In brief, ADSCs in the PTI/AP were fixed using a 2.5% glutaraldehyde solution for 15 min. Afterward, the PTI/AP was dehydrated in gradient ethanol and observed using an SEM (S4800, Hitachi, Japan).

The mechanical characteristics of the PTI/AP were subsequently studied. The uniaxial compression test of PTI/AP was performed using a universal mechanical testing machine with a probe speed of 0.5 mm/min, and the critical value was recorded. The elastic modulus was determined using the micro-indentation technique with a maximum load of 200 mN, an indentation rate of 400 mN/min, and a maximum penetration depth of 1,000 μm.

### 2.4 *In vitro* experiment

#### 2.4.1 Cell vitality, proliferation, and morphology assessment

ADSCs were seeded onto the blank cell wells as a control (CON), seeded in the PTIs, and incorporated into PTI/AP. After 3 days of culturing, the medium was removed, and the samples were stained in the dark upon adding calcein-AM/PI dye. The results were observed using a fluorescence microscope.

The CCK8 experiment was performed to investigate the effects of PTI/AP on cell proliferation. After ADSCs were cultured in the CON, PTI, and PTI/AP groups for 1, 3, and 7 days, the medium was changed, and then the CCK8 reagent at 10% of the medium volume was added. The absorbance of each group was measured at 450 nm after incubation in the dark for 2 h.

In order to observe cell morphology and spreading, the ADSCs in three groups were stained with phalloidin after 3 days of culturing. After the cells were fixed in a 4% paraformaldehyde and permeabilized with 0.1%Triton X-100, the F-actin was stained with rhodamine-labeled phalloidin reagent and the nucleus was stained with DAPI in the dark environment. The samples were observed using a fluorescence microscope. The F-actin density in all groups was evaluated using ImageJ software.

#### 2.4.2 Scratch assay and tube formation *in vitro*

The scratch assay and tube formation assay were evaluated to investigate the effect on angiogenesis *in vitro*. For the scratch assay, HUVECs were seeded in a 6-well plate for 24 h to form confluent monolayers. A small line was generated after gentle scraping with a sterile 200 μL pipette tip. The cell debris was washed with PBS, and CON, PTI, and PTI/AP were then co-cultured with the HUVECs. After the cells were cultured in an FBS-free RPMI1640 medium for 24 h, cell migration was observed, and the migration rate was calculated.

For tube formation assay, the matrigel matrix was added to 24 well plates at 289 μL/well on ice and incubated for 1 h at 37°C to obtain polymerized matrigel matrix. HUVECs were then seeded on a matrigel matrix and co-cultured with the scaffolds of the CON, PTI, and PTI/AP groups. After incubation for 6 h, tubular formation was observed using a light microscope, and the number of formed meshes was calculated using ImageJ software.

#### 2.4.3 Alkaline phosphatase (ALP) staining and activity evaluation

In order to study the effect of bioactive scaffolds on the osteogenic differentiation of ADSCs, ALP was detected. ADSCs in the CON, PTI, and PTI/AP groups were cultured for 7 days in an osteogenic induction medium, and ALP was stained using the BCIP/NBT method. Moreover, the ALP enzyme activity was evaluated. After 7 days of osteogenic induction, cells were lysed, and the lysate was incubated with pNPP chromogenic substrate (Beyotime, Shanghai, China) at 37°C for 30 min. The absorbance was detected at 405 nm.

#### 2.4.4 Alizarin red staining (ARS)

Extracellular matrix mineralization was studied using ARS. ADSCs cultured in the CON, PTI, and PTI/AP groups were fixed in 4% paraformaldehyde for 30 min after 7 and 14 days of osteogenic induction. Then, the cells were incubated with alizarin red dye at room temperature for 45 min. The staining results for each group were observed using a light microscope. Next, semi-quantitative analysis was performed by adding 10% cetylpyridinium chloride and measuring the absorbance at 540 nm.

#### 2.4.5 RT-qPCR

In order to study the effect on angiogenesis *in vitro*, expression levels of the angiogenesis-related genes HIF-1α and SDF-1α were detected by RT-qPCR. After 7 days of culture, the total RNA was extracted by using a centrifugation column purification kit. The isolated RNA was used to synthesize cDNA. Next, the target gene was amplified and detected. GAPDH served as an internal reference. In order to further study the effects on osteogenesis *in vitro*, expression levels of the osteogenesis-related genes RUNX2, ALP, and OCN were detected using the above methods by RT-qPCR. All primer sequences are shown in Supplementary Table S1.

#### 2.4.6 Immunofluorescence

The expression levels of osteogenic proteins were evaluated using immunofluorescence staining. After 7 days of osteogenesis induction, the cells were fixed in a 4% paraformaldehyde, then permeabilized with 0.1% Triton X-100 and blocked with 10% goat serum. After blocking, anti-Runx2 antibodies were added and incubated overnight at 4°C. Subsequently, FITC-labeled goat anti-rabbit antibody was added and incubated at 37°C for 1 h. After that, DAPI solution was added to stain the nucleus. The staining cells were observed using a fluorescence microscope, and the fluorescence intensity in each group was analyzed using ImageJ software.

### 2.5 *In vivo* evaluation

#### 2.5.1 Preparation of bone defects *in vivo* and implantation of the bioactive scaffolds

All experimental animal procedures were approved by the Animal Ethics Committee of Jilin University and complied with its established guidelines. A total of 28 5-month-old New Zealand rabbits were used in this study. A longitudinal incision was made in the left distal femur to expose the bony side of the femoral condyle. A cylindrical bone defect of 6 mm in diameter and 10 mm in depth was made using a bone drill. Then, PTIs, ADSCs directly loaded PTIs (PTI/A), PRP-filled PTIs (PTI/P), and PTI/AP were implanted, respectively, and the incisions were sutured layer by layer. All experimental animals were given intramuscular penicillin within 3 days of surgery to prevent infection.

#### 2.5.2 Micro-CT analysis

Micro-CT analysis (SkyScan 1076 scanner, Kontich, Belgium) was performed for *in vivo* bone regeneration studies 12 weeks postoperatively. The scanning voltage is 48 kv, the current is 12 μA, and the pixel size of the image is 18.26 μm. With the bone defect area as the region of interest (ROI), 3D reconstruction was performed. In addition, data analysis was performed, and the bone volume/total tissue volume ratio (BV/TV), bone mineral density (BMD), trabeculae number (Tb. N), and trabecular separation (Tb. Sp) of each ROI were calculated.

#### 2.5.3 Histological evaluation

To further evaluate osseointegration, femoral specimens were collected 12 weeks postoperatively and adequately fixed in 4% paraformaldehyde. These specimens were dehydrated using gradient ethanol and were embedded in methyl methacrylate. Then, hard tissue sections with a 30 μm thickness were cut from the specimens and stained using Van–Gieson (VG) staining and Masson’s trichrome staining. Quantitative analysis of bone area rates was performed using ImageJ software.

#### 2.5.4 Immunological evaluation

Immunofluorescence staining for CD31 and Runx2 proteins at the defect site was performed to assess angiogenesis and expression of osteogenic-related proteins. The collected femoral specimens were fixed in 4% paraformaldehyde, then decalcified, dehydrated, embedded in paraffin, and divided into 5 μm tissue sections. Then, the sections were incubated with anti-CD31 antibody and anti-Runx2 antibody at 4°C overnight. After that, sections incubated with anti-CD31 antibody were labeled with Cy3 goat anti-rabbit secondary antibody, while sections incubated with anti-Runx2 antibody were labeled with FITC goat anti-rabbit secondary antibody. The cell nucleus was stained with DAPI. The stained sections were observed using a fluorescence microscope, and the fluorescence intensity in each group was analyzed using ImageJ software.

### 2.6 Statistical analysis

Data from three independent experiments were expressed as mean ± standard deviation. The statistical comparison between groups was conducted using an unpaired two-tailed Student’s t-test. *p* < 0.05 was considered to be a significant statistical difference between groups.

## 3 Results and discussion

### 3.1 Characterization of the PTI/AP

**FIGURE 1:**
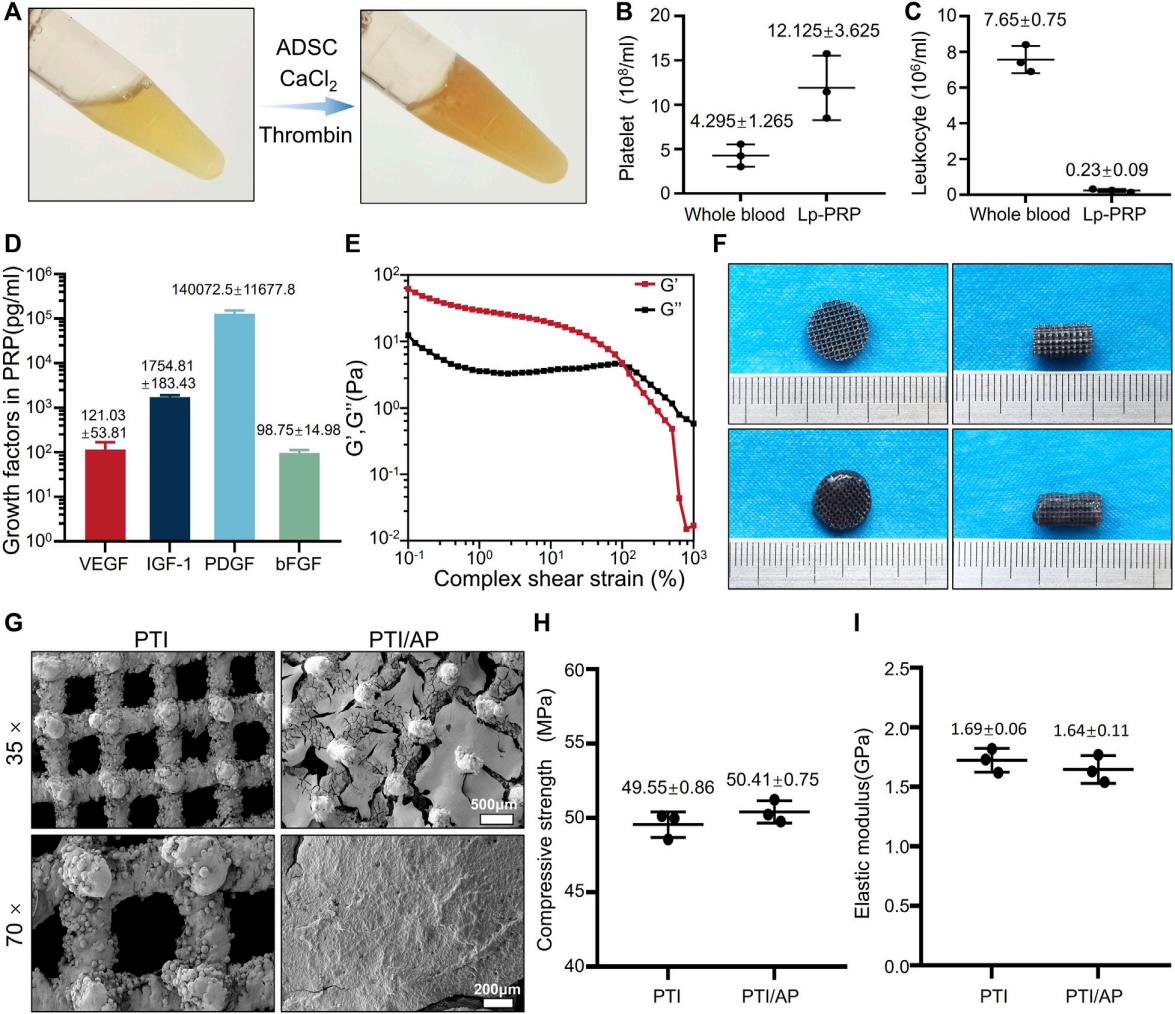
Characterization of the bioactive scaffold: **(A)**. Image of Lp-PRP gel; **(B, C)**: Platelet concentration **(B)** and leukocyte concentration **(C)** in Lp-PRP gel and whole blood samples; **(D)**. The concentrations of VEGF, IGF-1, PDGF and bFGF contained in Lp-PRP gel; **(E)**. Rheology properties of Lp-PRP gel; **(F)**. Appearance image of PTI, PTI/AP; **(G)**. SEM images of PTI and PTI/AP; **(H, I)**: Compressive strength **(H)** and elastic modulus **(I)** of PTI and PTI/AP.

In this study, Lp-PRP was obtained after three centrifugations. Compared with whole blood samples, Lp-PRP contains significantly enriched platelet content and negligible white blood cell content (Figures 1A–C). After thrombin and calcium chloride were added, the platelets in PRP released their granula contents, and PRP gel was obtained (Velier et al., 2018). The PRP gel contained high quantities of PDGF, IGF-1, FGF, and VEGF, which was consistent with previous studies (Figure 1D). These released growth factors can act on stem cells to promote their osteogenic differentiation and promote angiogenesis by acting on vascular endothelial cells, thus contributing to bone repair (Zhang et al., 2021; Liu et al., 2022). The rheological experiment results showed that the storage modulus (G′) of the PRP gel is higher than the loss modulus (G″) when the strain is less than 245%, and the PRP gel undergoes elastic deformation mainly to maintain a stable gel state. When the strain continues to increase, the G″ exceeds the G′, and the network structure in the PRP gel is damaged, and it changes from the gel to the solution state (Figure 1E). These results confirmed that the PRP gel was an appropriate carrier for stem cells. Cells can be mixed with PRP in a solution state, and the gelling process under a physiological state is conducive to maintaining cell activity. The relatively stable structure of the PRP gel can effectively retain the various growth factors and stem cells loaded therein *in situ*, which is crucial for treatment. Hydrogels have customizable structure, good biocompatibility and adjustable mechanical strength, which can promote the transport of nutrients, cell migration and proliferation, and finally promote osteogenic differentiation (Li et al., 2021; Zhao et al., 2022).

Based on these results, ADSCs were loaded into the PRP gel and modified on the surface of PTIs. In fact, because the cell culture medium contains CaCl_2_, it also promotes the gelation of liquid PRP. After the mixed solution fully fills the pores of the scaffold, it becomes a gel state and no longer flows. (Figure 1F). Our experiment confirmed that the extracted ADSCs had good osteogenic, chondrogenic, and adipogenic differentiation capabilities (Supplementary Figure S1A) and exhibited typical stem cell surface antigen characteristics of CD34^−^, CD45^−^, and CD90^+^ (Supplementary Figure S1B). As the most commonly used material for orthopedic implants, titanium alloy has high mechanical strength and corrosion resistance, but its high stiffness limits its application, which leads to stress shielding-induced osteolysis. 3D printed porous titanium alloy scaffold can significantly reduce the stiffness and accurately manufacture customized prostheses with complex shapes and structures. The microporous structure can increase the contact area, and these shapes and surface areas have interconnected pores, which promotes bone ingrowth, thus facilitating bone integration (Bai et al., 2020a; Li et al., 2022a). PTIs with a porosity of 70% were prepared using 3D printing (Figure 1F). SEM detection showed that PTIs had an interconnected pore structure, and their pore size was 602.66 ± 11.08 μm (Figure 1G). The structural features of the micropores are bionic designs based on the normal cancellous bone structure, which can increase the bone conductivity of the material and promote bone ingrowth (Cui et al., 2021). Different porosities and pore sizes produce different cell response effects. Previous studies have shown that a pore size of 300–500 μm and a porosity of approximately 75%–90% is ideal, which is close to the pore characteristics of human cancellous bone structure (Wang et al., 2017; Zhou et al., 2022). PTIs designed in this study have a slightly larger pore size for better PRP gel loading.

Subsequently, PTI/AP scaffolds were successfully constructed. Based on SEM images, it can be observed that there were well-formed ADSCs on the surface of PTI/AP. (Figures 1F, G). The mechanical properties studies results showed that the compressive strengths of PTIs and PTI/AP were 49.55 ± 0.86 and 50.41 ± 0.75 MPa, and the elastic moduli were 1.69 ± 0.06 and 1.64 ± 0.11 GPa (Figures 1H, I). The compressive strength and elastic modulus of human cortical bone are in the range of 100–250 MPa and 7–20 GPa, respectively. The corresponding values for cancellous bone are in the range of 11–24 MPa and 1.5–11.2 GPa, respectively (Kapat et al., 2017). The PTI/AP in the present study has mechanical properties similar to normal bone tissue so that stress shielding and osteolysis after material implantation can be effectively avoided (Bai et al., 2020a).

### 3.2 Biocompatibility, cell proliferative activity, and cell morphology of PTI/AP

**FIGURE 2:**
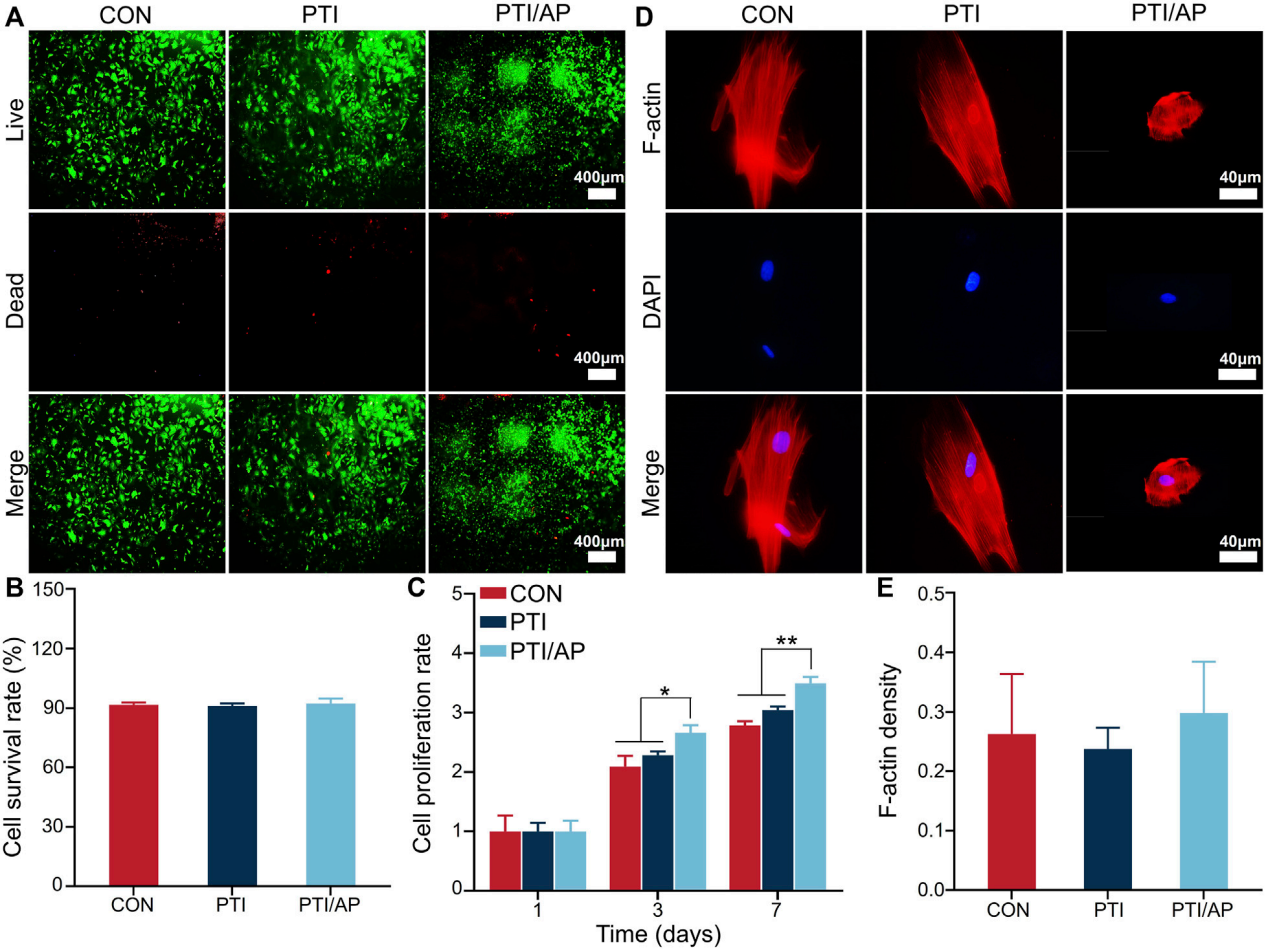
Evaluation of cell viability, proliferation and morphological of encapsulated ADSC in PTI/AP: **(A)**. Calcein-AM/PI staining after 3 days; **(B)**. Cell survival rate in each group based on calcein AM/PI staining; **(C)**. CCK8 results; **(D)**. F-actin/DAPI staining of ADSC after 3 days; **(E)**. The semi-quantitative statistics of F-actin density of ADSC. (*n* = 3, *p < 0.05, **p < 0.01).

As shown in Figure 2A, the number of living cells in all three groups was significantly higher than the number of dead cells. Based on the staining results, we quantified the cell survival rate using ImageJ. The cell survival rates in the CON, PTI, and PTI/AP groups exceeded 90% without a statistical difference (Figure 2B). The CCK8 experiment results are shown in Figure 2C. From day 1 to day 7, cells in all three groups exhibited a gradual proliferation trend. The cell proliferative activities of the PTI/AP group were significantly higher than the other two groups on days 3 and 7. Previous studies have shown that various growth factors in the PRP matrix can promote cell proliferation. In addition, the porous structure of the 3D printing scaffold also provides sufficient space for cell proliferation (Bai et al., 2020a; Sharun et al., 2021). The limited number and survival rate of transplanted cells has always been a challenge for stem cell therapy for bone defects (Whitely et al., 2018). PTI/AP not only provides a stable carrier for transplanted cells but also maintains their activity and promotes their proliferation.

F-actin was stained with rhodamine–phalloidin to study the cell morphology of the ADSCs in each group. The cells on PTIs showed spindle morphology similar to that of the CON group. However, in the PTI/AP group, the ADSCs located in the PRP gel matrix exhibited a spherical shape because the cells were uniformly surrounded by a hydrogel with a set strength, which also allowed them to form 3D spatial contact with the PRP matrix components. These spherical cells had intact nuclei and abundant F-actin that was clearly visible (Figure 2D). Quantitative analysis also revealed that the ADSCs encapsulated in PRP matrix in the PTI/AP group had similar F-actin density compared with the CON and PTI groups (Figure 2E). As F-actin is a key component in the cytoskeleton, it plays an important role in the function and maturation of cells, and even in early osteogenic differentiation (Thomas et al., 2002; Li et al., 2019). This further confirmed that the cells in the PTI/AP group had a good growth and differentiation state.

### 3.3 PTI/AP drives angiogenesis of HUVECs *in vitro*

**FIGURE 3:**
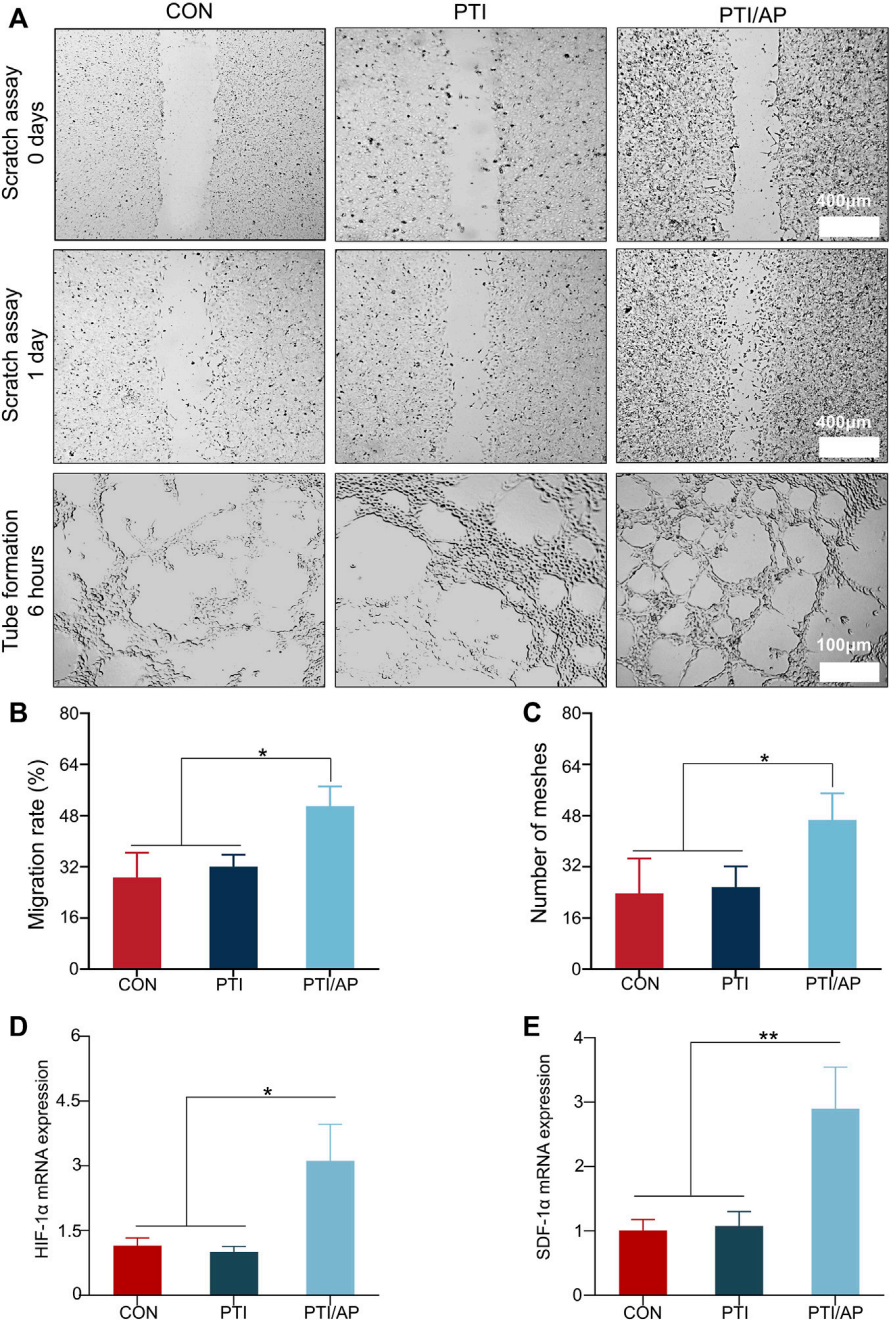
Effect on angiogenesis *in vitro*: **(A)**. The results of scratch assay after HUVEC were cultured with CON, PTI, PTI/AP group for 24 h and the tubular formation *in vitro* after 6 h; **(B)**. Cell migration rate of HUVEC in each group; **(C)**. Semi-quantitative analysis of mesh number in tubule formation experiment. **(D, E)**. Relative expression levels of HIF-1α **(D)** and SDF-1α **(E)**. (*n* = 3, *p < 0.05, **p < 0.01).

The effects of PTI/AP on HUVEC cell migration were evaluated using a scratch test. Figure 3A shows that the number of cells migrating to the scored area significantly increased in the PTI/AP group. The quantitative evaluation also confirmed that the migration rate was highest in the PTI/AP group (Figure 3B) (*p* < 0.05).

The tubule formation test was used to analyze the angiogenesis potential of PTI/AP. Most tubes begin to be formed in 2–4 h, and the peak number of tubes is between 3–12 h (DeCicco-Skinner et al., 2014). We chose 6 h as the best observation time. As shown in Figure 3A, the initial dense HUVEC layers in the PTI/AP group aligned into more organized tubular cell structures with many intact annular vascular conformations observed after 6 h of culturing. However, delayed tubular formation was exhibited in the CON and PTI groups, with only a few tubular cell organizations observed. The number of meshes formed in the PTI/AP group was significantly higher than in the other two groups (Figure 3C) (*p* < 0.05).

In order to further study the effect of PTI/AP on angiogenesis, we studied the expression of angiogenesis-related genes. HIF-1α mediates the response of cells to changes in oxygen levels and controls physiological and pathological angiogenesis, which is an important transcription factor related to angiogenesis (Li et al., 2022b). The relative expression level of HIF-1α in PTI/AP group was 2.95 ± 0.79, which was significantly higher than that in CON group (0.98 ± 0.12) and PTI group (1.20 ± 0.16). The expression of HIF-1α is shown in Figure 3D. When HIF-1α signaling pathway is activated, it can start the expression of its downstream gene SDF-1α (also known as CXCL12), which is the main regulator of vascular growth (Mehrabani et al., 2015; Ha et al., 2022). As shown in Figure 3E, the expression levels of SDF-1α in CON, PTI and PTI/AP groups were 1.02 ± 0.17, 1.14 ± 0.20 and 2.79 ± 0.62, respectively, which indicated that the expression of SDF-1α in PTI/AP group was significantly upregulated.

These results confirmed that the PTI/AP group promoted angiogenesis *in vitro*. This is also consistent with previous studies in which PRP gels effectively induced the proliferation and migration of endothelial cells (Wei et al., 2021; Guo et al., 2017; do Amaral et al., 2019). PRP gel contains abundant angiogenesis-related growth factors such as PDGF, IGF-1, bFGF, and VEGF. PDGF could promote the migration and angiogenesis of HUVECs by activating the PI3K/AKT and ERK1/2 signaling pathways (Zhang et al., 2018). IGF-1 can also upregulate endothelial nitric oxide synthase activity (Borselli et al., 2010; Friedrich et al., 2018). Furthermore, bFGFs are an effective mitogen inducer in endothelial cells (Guo et al., 2006). The release of these growth factors from PTI/AP promoted angiogenesis and thus provided the cells and nutrients necessary for bone defect repair.

### 3.4 Enhanced osteogenic differentiation in PTI/AP *in vitro*

**FIGURE 4:**
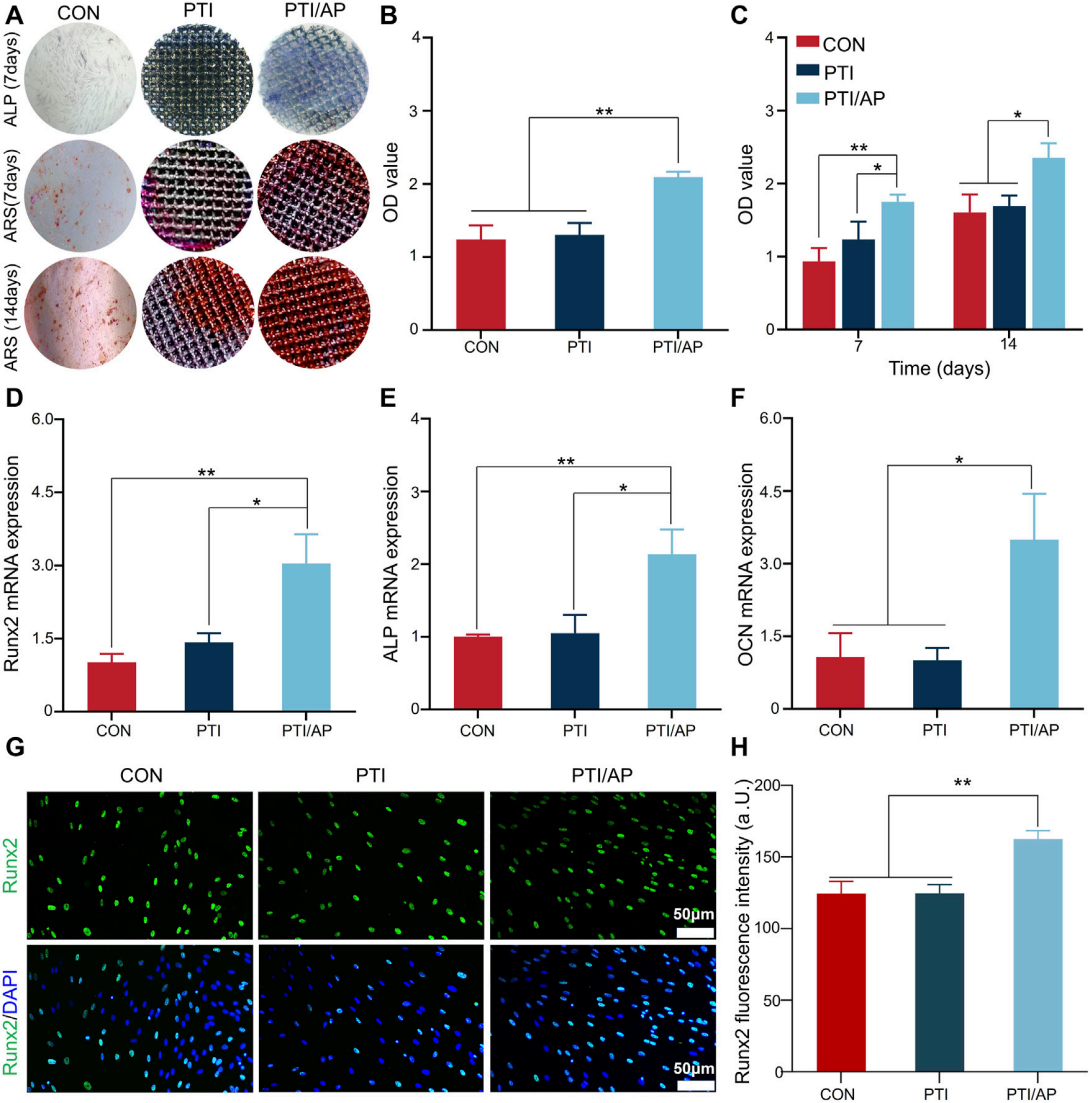
Effect on osteogenic differentiation of encapsulated ADSC: **(A)**. ALP staining of ADSC after 7 days of osteogenic induction and ARS after 7 days and 14 days of osteogenesis induction; **(B)**. ALP enzyme activity of ADSC after 7 days of osteogenic induction; **(C)**. Semi-quantitative analysis of ARS staining; **(D–F)**: Relative expression levels of Runx2 **(D)**, ALP **(E)** and OCN **(F)**; **(G)**. Immunofluorescence staining of Runx2; **(H)**. Semi-quantitative analysis of fluorescence intensity of Runx2. (*n* = 3, *p < 0.05, **p < 0.01).

The effect of PTI/AP on the osteogenic differentiation was studied using the ALP assay and ARS. ALP is one of the important markers of osteogenic differentiation, which is mainly involved in the early process of extracellular matrix mineralization (Zhou et al., 2022). As shown in Figure 4A, the PTI/AP group exhibited the highest amount of purple ALP staining product after 7 days. The ALP enzyme activity test showed that the ALP enzyme activity of the PTI/AP group was the highest (Figure 4B).

Calcium and phosphate deposition and extracellular matrix mineralization are the hallmarks of mature osteoblasts (Murshed, 2018). In this study, we studied the mineralized calcium nodule content through the ARS experiment. Gross images showed limited stained calcium nodules on day 7 in both the CON and PTI groups, while significantly more calcium nodules were observed in the PTI/AP group, and the trend was more pronounced on day 14. Subsequent semi-quantitative analysis also revealed greater amounts of calcium nodules on both day 7 and day 14 in the PTI/AP group compared with the CON and PTI groups (Figures 4A, C).

In order to further study the effect of PTI/AP on osteogenic differentiation, we studied the expression of osteogenic-related genes and proteins. Runx2 is an early sign of osteogenic differentiation (Komori, 2019). The expression of Runx2 is shown in Figure 4D. The relative expression level of Runx2 in the PTI/AP group was 3.04 ± 0.60, which was significantly higher than the CON (1.01 ± 0.17) and PTI groups (1.42 ± 0.19). A higher ALP expression level of 2.14 ± 0.34 was observed in the PTI/AP group, while the expression levels in the CON and PTI groups were 1.01 ± 0.03 and 1.04 ± 0.25 respectively (Figure 4E). OCN is the most abundant non-collagen protein in the extracellular matrix, which is a key marker of mature osteoblast (Komori, 2020). As shown in Figure 4F, the expression levels of OCN in the CON, PTI, and PTI/AP groups were 1.06 ± 0.50, 1.01 ± 0.25, and 3.49 ± 0.95 respectively, which indicated that the expression of OCN in PTI/AP group was significantly upregulated.

The expression of Runx2 at the protein level was studied using immunofluorescence. As shown in Figure 4G, compared with the CON and PTI groups, more green fluorescent areas and positive cell amounts were observed in the PTI/AP group. Quantitative analysis showed that the average fluorescence intensity of the PTI/AP group was significantly higher than in the CON and PTI groups (Figure 4H).

Our results demonstrated that PTI/AP can promote the osteogenic differentiation of encapsulated ADSCs. Growth factors in the PRP gel contribute to this result. Zhang et al. (2018) confirmed that PDGF increased osteogenic differentiation but inhibited adipogenic differentiation of BMSCs via the ERK1/2 signaling pathway. IGF1 and bFGFs are also growth factors that promote osteogenesis (Chen et al., 2011; Friedrich et al., 2018). Therefore, our *in vitro* results confirmed that the constructed PTI/AP bioactive scaffold has the dual function of promoting angiogenesis and osteogenic differentiation, so it has the appropriate ability to promote bone defect repair.

### 3.5 Promoted bone regeneration in PTI/AP *in vivo*

Because the local bone tissue of rabbits does not contain ADSCs and PRP, we added PTI/A group and PTI/P group to the experimental group *in vivo* to observe the relationship between ADSCs and PRP on local bone regeneration and vascular regeneration in rabbits.

**FIGURE 5:**
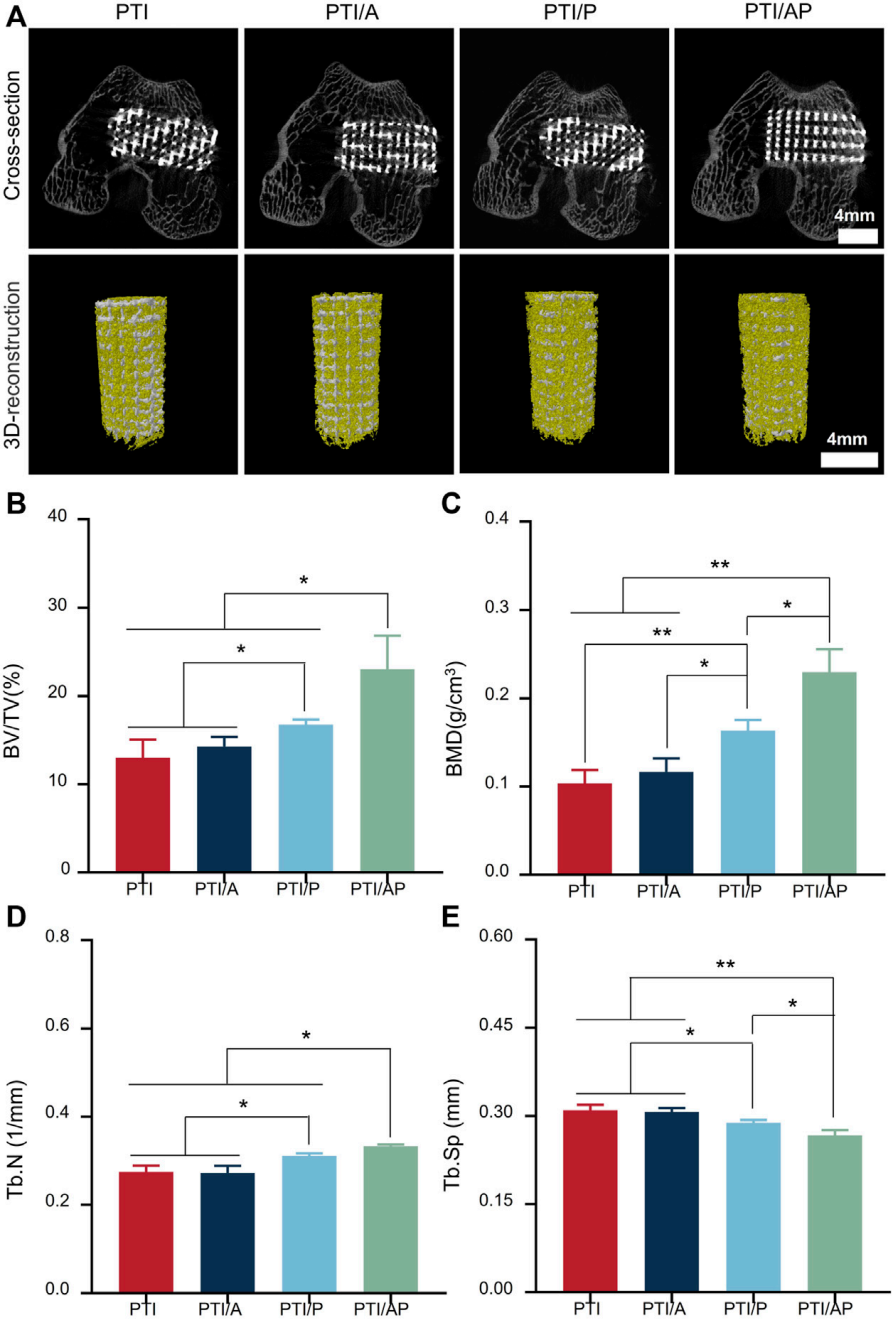
Micro CT evaluation for bone repair: **(A)** Representative cross-sectional images and 3D reconstruction images after the implantation of PTI, PTI/A, PTI/P, and PTI/AP into femoral defects for 12 weeks; **(B–E)**: BV/TV, **(B)** BMD **(C)**, Tb.N **(D)**, Tb. Sp **(E)** in the defect site. (*n* = 3, *p < 0.05, **p < 0.01).

The distal femoral defect was successfully established to study the effect of PTI/AP on promoting bone regeneration *in vivo*. At 12 weeks after the operation, micro-CT scanning was used to evaluate the new bone regeneration and vascular regeneration. As shown in Figure 5A, bone regeneration can be observed in all groups in both cross-section view and 3D reconstruction images. Compared with the PTI, PTI/A, and PTI/P groups, the PTI/AP group has the highest amount of new bone. From the cross-section view, it could be seen that the internal PTI/AP pores also had significant new bone regeneration, whereas the new bone mass in the other three groups was limited. Data analysis of the scanning area showed that the BV/TV of the PTI/AP group was 23.81% ± 3.01%, which was significantly higher than the PTI (12.99% ± 2.08%), PTI/A (14.28% ± 1.09%), and PTI/P groups (16.75% ± 0.58%). Moreover, the BV/TV of the PTI/P group was significantly higher than the PTI and PTI/A groups (Figure 5B). Similarly, the BMD and Tb.N values of the PTI/AP group were significantly higher than the PTI and PTI/A groups and slightly higher than the PTI/P group. The PTI/P group also had a significantly higher value than the PTI and PTI/A groups (Figures 5C, D). The Tb. Sp of the PTI/AP and PTI/P groups is lower than the PTI and PTI/A groups, and the PTI/AP group is the lowest (Figure 5E).

**FIGURE 6:**
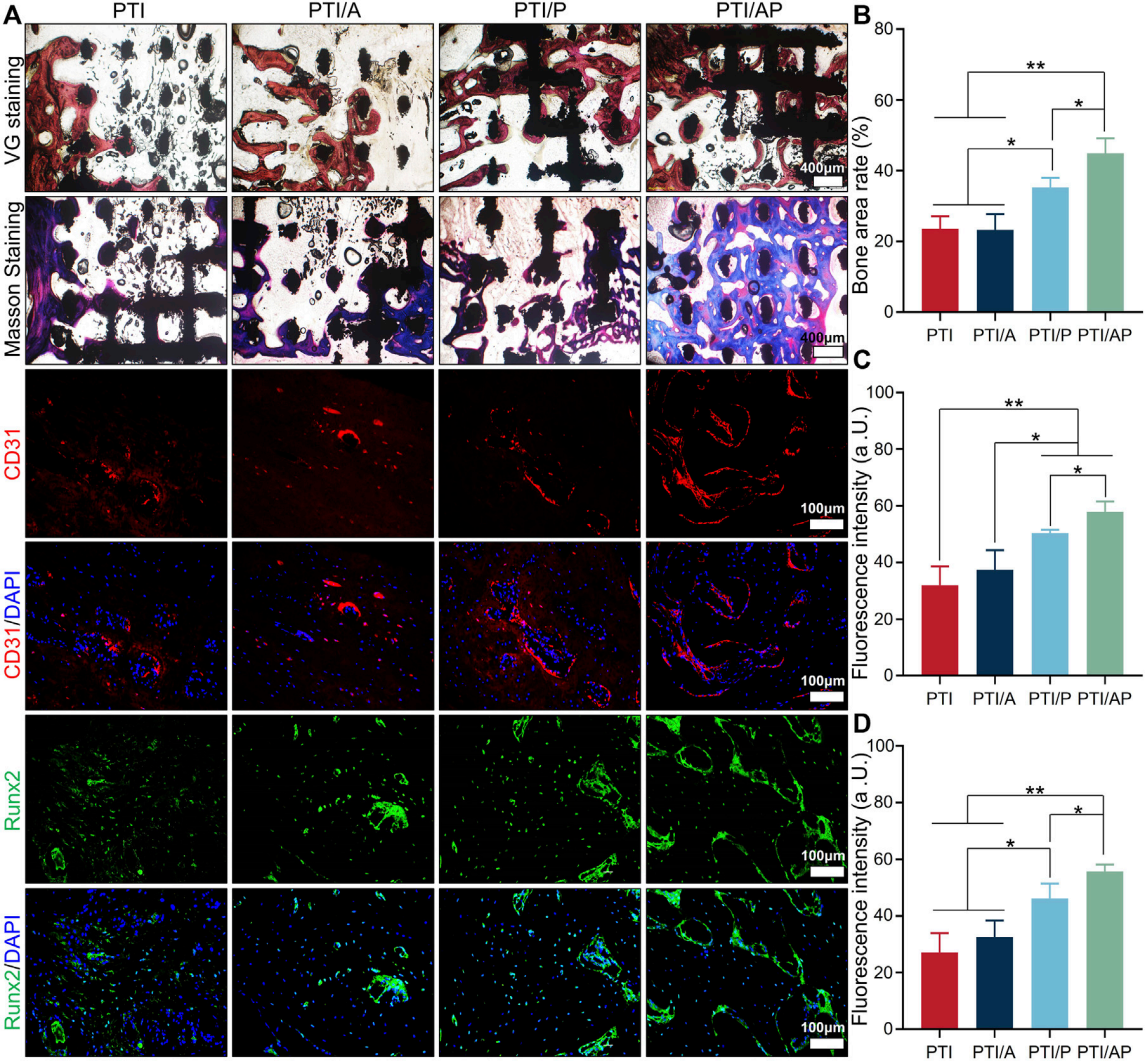
Histological and immunological analysis of bone regeneration: **(A)**. Masson staining, VG staining, CD31 and Runx2 immunofluorescence staining at 12 weeks; **(B)**. Semi-quantitative analysis of bone regeneration area based on VG staining; **(C, D)**: Fluorescence intensity based on CD31 staining **(C)** and Runx2 staining **(D)**. (*n* = 3, *p < 0.05, **p < 0.01).

Representative Masson and VG staining images are presented in Figure 6. At 12 weeks, there was little bone regeneration at the interface between the scaffold and the host bone in both the PTI and PTI/A groups, and newly formed bone tissue was barely visible inside the scaffold. In the PTI/P group, new bone regeneration can be seen at the interface between the scaffold and the host bone and inside the scaffold, and its bone repair is superior compared with the PTI and PTI/A groups. In the PTI/AP group, a considerable amount of new bone can be observed both at the interface of the scaffold and host bone and inside the scaffold. Semi-quantitative analysis based on VG staining also supported the staining results. The new bone mass in the PTI/AP and PTI/P groups was significantly larger than the PTI and PTI/A groups, and the new bone mass in the PTI/AP group was the largest (Figure 6B). Semi-quantitative analysis based on Masson staining also showed similar results (Supplementary Figure S2).

Immunofluorescence was used to study the angiogenesis and the expression of osteogenic-related proteins in the defect site. CD31 is well recognized as a marker of vascular endothelial cells (Sass et al., 2017). As shown in Figure 6, the red area of CD31 fluorescent staining cells in the PTI/AP and PTI/P groups was significantly higher than the PTI and PTI/A groups; the PTI/AP group was the highest. The semi-quantitative analysis also results confirmed this conclusion (Figures 6A, C). The immunofluorescence staining results of osteogenesis-related protein Runx2 showed that the green positive staining area of the defect site in the PTI/AP and PTI/P groups was significantly larger than the PTI and PTI/AP groups, and the PTI/AP group was larger than the PTI/P group. The semi-quantitative analysis of fluorescence intensity showed the same trend, namely, PTI/AP > PTI/P > PTI and PTI/A (Figures 6A, D).

Bone defect has always been a difficult problem in orthopedics. The treatment of bone defects mainly includes two primary missions, namely, ensuring mechanical stability and improving local bone repair ability (Song et al., 2017; Peng et al., 2021). In this study, a scaffold with pore structure and mechanical characteristics similar to normal bone was prepared, which provided a mechanically stable microenvironment for the defect site. For improved bone repair ability, this study used PRP gel as a carrier, loaded with ADSCs combined with PTIs to construct a dual-functional bioactive scaffold that can promote osteogenesis and angiogenesis. After 12 weeks of implantation into the femoral defect of rabbits, it was confirmed that the PRP gel supported the cell viability, migration, and differentiation of the encapsulated ADSCs, and the growth factors in the PRP matrix promoted vascular regeneration and bone formation, thus accelerating bone defect repair. It is worth noting that the PTI/A group did not have a significant treatment effect. This proves that in the absence of appropriate carriers and effective nutritional supply, it is difficult for transplanted cells to maintain their activity and cannot exert therapeutic effects. Studies have shown that in order to maintain the activity of seeded cells, the distance between cells and blood vessels should not exceed 200 μm (Kaully et al., 2009). Therefore, the speed and degree of vascularization play a vital role in the efficiency of tissue engineering materials and bone regeneration. In the PTI/AP group, the existence of the PRP gel enables the encapsulated ADSCs to remain in the defect site and maintain their activity, and various growth factors in it promote the growth of new blood vessels, thus providing necessary nutrients for ADSCs.

Moreover, the PTI/P group played a better role in angiogenesis and bone repair than the PTI and PTI/AP groups by releasing various growth factors into the PRP gel, but its effect was not as good as the PTI/AP group. Some previous literature studies also reported that a combination of BMSCs and PRP enhanced new bone formation and vascularization and promoted bone defect repair in dogs and rabbits, while the PRP alone group achieved unsatisfactory bone repair (Nair et al., 2009; Yoshimi et al., 2009). The interaction between the osteogenic differentiation of stem cells and the angiogenesis of endothelial cells could result in this difference. ADSCs could produce growth factors such as VEGF, FGF, and hepatocyte growth factors that are essential for the migration and proliferation of endothelial cells (Zhang et al., 2018; Murohara, 2020). Moreover, ADSCs can differentiate into endothelial cells under appropriate conditions, thus directly participating in vascular regeneration. In addition, ADSCs can also be used as peripheral cells to support and stabilize the nascent endothelial cells (Traktuev et al., 2008; Ebrahimian et al., 2009). Similarly, endothelial cells not only participate in angiogenesis but also produce factors that promote osteogenesis, such as bone morphogenetic protein (Wang et al., 2011; Man et al., 2012).

## 4 Conclusion

Overall, we constructed a PTI/AP bioactive scaffold by filling ADSC-loaded PRP gel into PTI pores. Our experimental results demonstrated that this bioactive scaffold had suitable mechanical properties and cell compatibility. Abundant growth factors were present in the PRP gel matrix, which maintained the cell activity and morphology, promoted the proliferation and osteogenic differentiation of encapsulated ADSCs, and regulated the migration of endothelial cells and angiogenesis, thus promoting bone defect repair in the distal femur. Therefore, this dual-function scaffold with osteogenesis and angiogenesis provides a promising strategy for clinical treatment of bone defects.

## 5 Materials

The Ti6Al4V powders (Grade 23, particle size 45–100 μm) were purchased from AK Medical Co., Ltd (Beijing, China). Thrombin, calcium chloride, and phosphate buffers (PBS) were provided by Yuanye Bio-Technology Co., Ltd (Shanghai, China). Collagenase type I, Dulbecco’s Modified Eagle Medium/Nutrient Mixture F-12 (DMEM/F-12), trypsin-EDTA (0.25%), fetal bovine serum (FBS), Roswell Park Memorial Institute (RPMI) 1,640 medium and streptomycin–penicillin were purchased from Gibco Life Technology (CA, United States); Enzyme-linked immunosorbent assay (ELISA) kits for PDGF, IGF-1, bFGF and VEGF were purchased from Jianglai Biotechnology Co., Ltd (Shanghai, China). Cell Counting Kit 8 (CCK-8) and Calcein-AM/PI were purchased from Beyotime Biotechnology (Shanghai, China). Osteogenic medium for rabbit ADSCs and alizarin red staining (ARS) dye, human umbilical vein endothelial cells (HUVEC) were purchased from Cyagen Biosciences (Suzhou, China). Total RNA extraction kit, cDNA synthesis kit and PCR amplification kit, anti-Runx2 antibody, and Cy3 goat anti-rabbit secondary antibody were got from ABclonal Biotechnology (Wuhan, China). FITC goat anti-rabbit secondary antibody anti-CD31 antibody was got from Bioss Biotechnology (Beijing, China).

## 6 Isolation and characterizations of ADSC

Allogeneic adipose-derived stem cells were extracted from subcutaneous adipose tissue of rabbit inguinal region according to previous methods (Yuksel et al., 2000). In brief, adipose tissues were minced into small pieces and then incubated in 0.1% collagenase type I at 37°C for 90 min. The resulting cell suspension was subjected to filtration and centrifugation at 1,200 rpm for 10min. The cell pellet was re-suspended and cultured in DMEM/F12 medium supplemented with 10%FBS and 1% streptomycin-penicillin.

To verify the stem cell characteristics of the obtained ADSC, its osteogenic, chondrogenic, and adipogenic differentiation abilities were detected via ARS, alcian blue staining and oil red O staining respectively. The surface antigens of ADSC were identified by flow cytometry. The cultured cells were fixed with 4% paraformaldehyde, and blocked with 10% goat serum. Subsequently, anti-CD34, anti-CD45 and anti-CD90 antibodies were added and incubated respectively. After washing, FITC-labeled goat anti-rabbit secondary antibody was added and incubated for 1 h and then detected by flow cytometry.

## Data Availability

The original contributions presented in the study are included in the article/Supplementary Material, further inquiries can be directed to the corresponding authors.
