# Supplementary material for: Engineered three-dimensional bioactive scaffold for enhanced bone regeneration through modulating transplanted adipose derived mesenchymal stem cell and stimulating angiogenesis
Source: Front Bioeng Biotechnol. 2024 Jan 26;12:1342590. doi: 10.3389/fbioe.2024.1342590 (PMC10853357; doi:10.3389/fbioe.2024.1342590)
Supplement: Supplementary file 1 [file Table1.DOCX]

**Table S1**. **The primer sequences in this study**

| **Gene subtype** | **Oligonucleotide Primers (5’-3’)** |
| --- | --- |
| HIF-1α | F: 5′-ACCGCTGAAACGCCAAAG-3′  R: 5′-TCCATCGGAAGGACTAGGTGTCT-3′ |
| SDF-1α | F: 5′- ACCGCGCTCTGCCTCAGCGACGGGAAG-3′  R: 5′- TGTTGTTCTTCAGCCGGGCTACAATCTG-3′ |
| GAPDH | F: 5′- ATTGACCTCAACTACATGGTTTACATG-3′  R: 5′- TTGGAGGGATCTCGCTCCTGGAAG-3′ |

| **Gene subtype** | **Oligonucleotide Primers (5’-3’)** |
| --- | --- |
| RUNX2 | F: 5′- TCAGGCATGTCCCTCGGTAT -3′  R: 5′- TGGCAGGTAGGTATGGTAGTGG -3′ |
| ALP | F: 5′-GCACTCCCACTTTGTCTGGA-3′  R: 5′-TCCTGTTCAGCTCGTACTGC-3′ |
| OCN | F: 5′- CAGAGCGACAGCATGAGGG-3′  R: 5′- CCTCTGCCAGACTCTGCACC-3′ |
| GAPDH | F: 5′- GTTCCACGGCACGGTCAAGG-3′  R: 5′- CCAGGGGGGCTAAGCAGTTG-3′ |
