# Supplementary figures and images for: Engineered three-dimensional bioactive scaffold for enhanced bone regeneration through modulating transplanted adipose derived mesenchymal stem cell and stimulating angiogenesis
Source: Front Bioeng Biotechnol. 2024 Jan 26;12:1342590. doi: 10.3389/fbioe.2024.1342590 (PMC10853357; doi:10.3389/fbioe.2024.1342590)

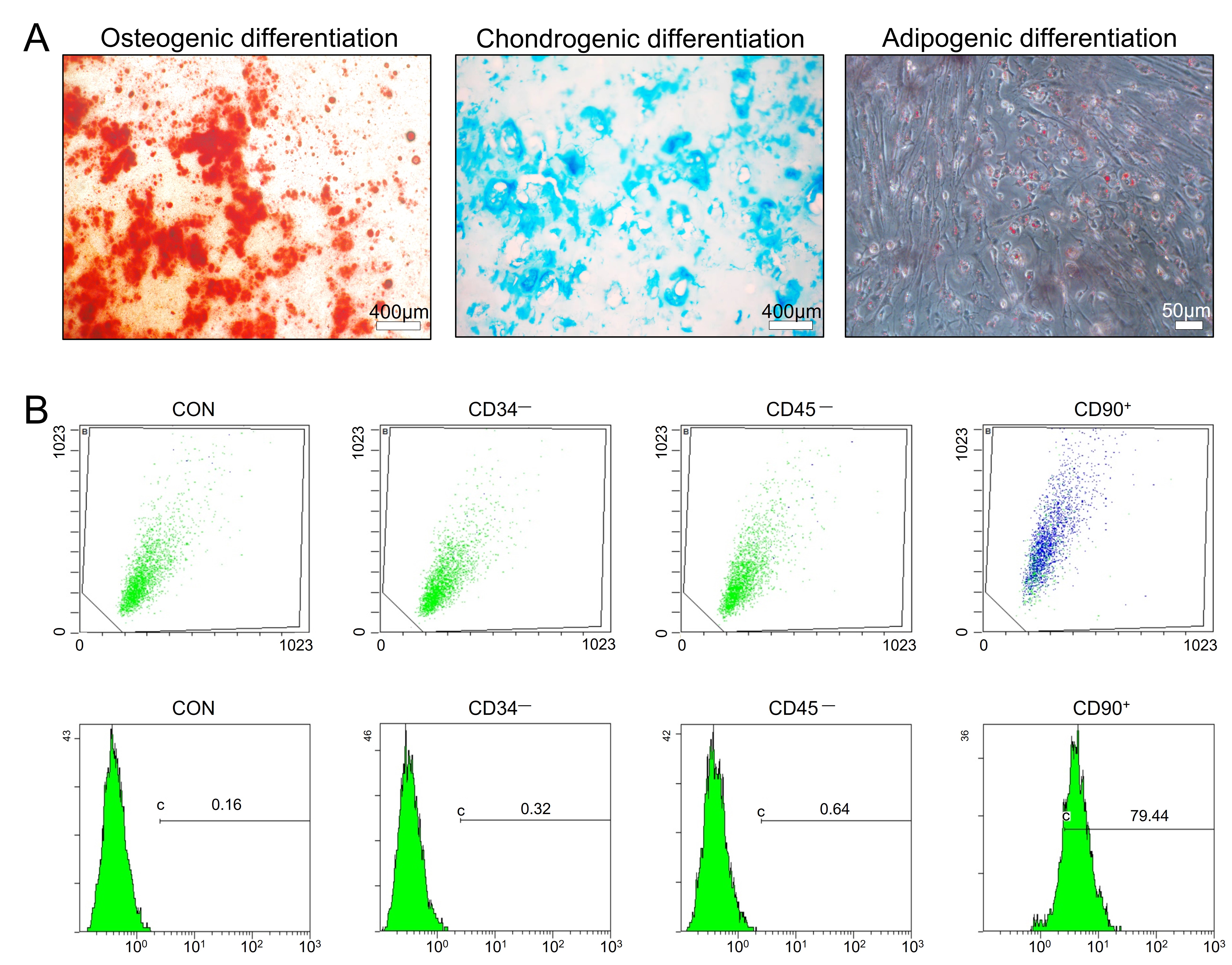

Supplement: Supplementary file 2 [file Image1.JPEG]

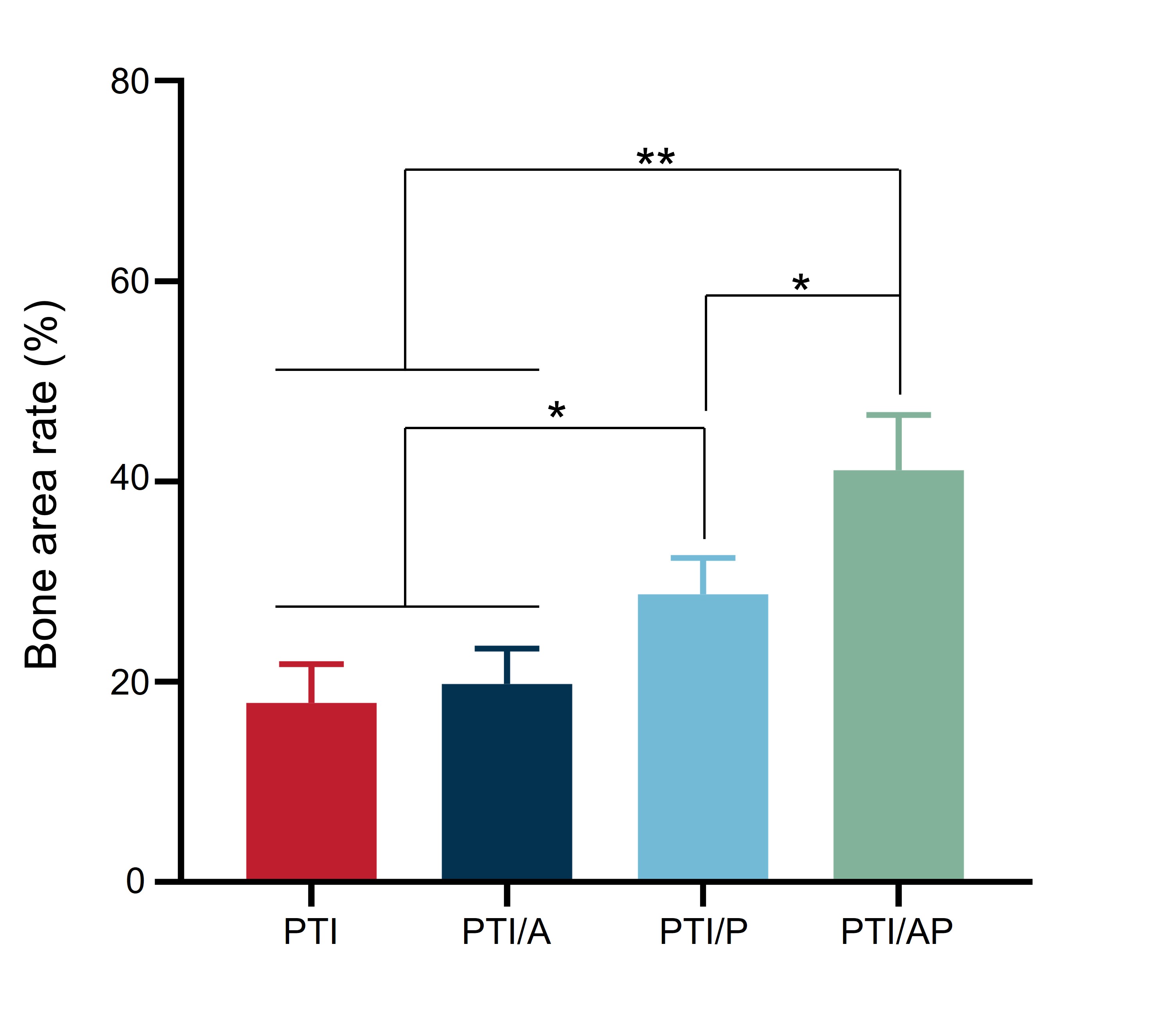

Supplement: Supplementary file 3 [file Image2.JPEG]
